# Mapping encounters between Antarctic krill fishing vessels and air-breathing krill predators using acoustic data from the fishery

**DOI:** 10.1073/pnas.2417203122

**Published:** 2025-06-16

**Authors:** Dominik Bahlburg, Sebastian Menze, Bjørn A. Krafft, Andy D. Lowther, Bettina Meyer

**Affiliations:** ^a^Polar Biological Oceanography, Alfred Wegener Institute, Helmholtz Center for Polar and Marine Research, Bremerhaven 27570, Germany; ^b^Helmholtz Institute for Functional Marine Biodiversity, Oldenburg 26129, Germany; ^c^Institute of Marine Research, Bergen 5005, Norway; ^d^Norwegian Polar Institute, Tromsø 9296, Norway; ^e^Institute for Chemistry and Biology of the Marine Environment, Oldenburg 26129, Germany

**Keywords:** Antarctic krill, fishery, predator encounters, acoustic, convolutional neural network

## Abstract

Antarctic krill is a keystone species in the Antarctic marine ecosystem and the target of a growing fishery. Given the ecological importance of krill, concerns have been raised about potential negative impacts of fishing on the Southern Ocean ecosystem. Resource-efficient approaches to fisheries monitoring are particularly valuable in this context due to the high costs associated with data collection in Antarctica. In this study, we trained a segmentation model (U-Net) to extract dives of air-breathing krill predators from more than 30,000 h of active acoustic data collected by three krill fishing vessels over six years. We were able to characterize the temporal and spatial dynamics of predator-vessel co-occurrences, which aligned well with the findings from more costly tracking studies. For example, we found that encounters with whales consistently peaked in autumn around the Antarctic Peninsula, when whales are building up fat reserves for their migration to breeding grounds. We also demonstrated that protection measures, introduced to protect breeding penguins at the Antarctic Peninsula, have simply shifted penguin-vessel encounters to the South Orkney Islands, where the affected colonies are not currently monitored. Our approach, results, and application example demonstrate how acoustic data from fishing vessels can provide important information to support fisheries management. As a by-product of fishing operations, these data are cost-effective, offering unique temporal and spatial coverage and providing a useful basis for rapid, low-level assessments of the fishery’s interaction with the wider ecosystem. This is particularly important given the unpredictable dynamics of krill fishery management decision-making.

## Significance

Antarctic krill is critical to the Antarctic ecosystem and the target of a growing fishery. To minimize the impact of fishing on the ecosystem, cost-effective monitoring is essential. In this study, we developed a method to detect krill predators diving beneath fishing vessels. This allowed us to analyze predator-vessel encounters at very large scales. We found that the seasonality and distribution of encounters were highly predator-specific and that existing protection measures were ineffective in protecting breeding penguins during the summer. We therefore not only provide insights into the overlap of predators and vessel in the krill fishery but also present an approach to data collection that can inform fisheries management at low cost.

The fishery for Antarctic krill (*Euphausia superba*, hereafter referred to as “krill”) is one of the world’s largest fisheries for crustaceans (1), and by far, the largest fishery in the Southern Ocean (2). The key fishing areas, namely South Georgia, the South Orkney Islands and the Antarctic Peninsula, are among the most productive regions in the Southern Ocean, home to millions of penguins, seals, and large populations of baleen whales. These and many other Southern Ocean species are critically dependent on krill as a food source, making it a keystone species (3–6). Understanding and mitigating the impacts of fishing on krill populations and their predators is therefore key to preserving the important functions that krill provide to the Southern Ocean ecosystem.

## Ecosystem Impacts of the Krill Fishery.

The Convention for the Conservation of Antarctic Marine Living Resources (CCAMLR) manages the krill fishery using an ecosystem approach that aims to avoid negative impacts not only on the target species (krill) but also on dependent taxa. However, increasing catches (2), recovering whale populations (7), an increasing spatial concentration of the fishing activity (8), and ecosystem change due to climate warming (9) raise concerns about potential detrimental effects of the fishery on the Southern Ocean ecosystem (10–12). In addition, the CCAMLR conservation measure 51-07 which spread the total catch limit of 620,000 tonnes across smaller management subareas, expired in October 2024. As a result, the fishery is currently free to decide where to fish within the CCAMLR area 48, which might be in conflict with CCAMLR’s precautionary principle as it increases the risk of further spatial concentration of catches and ecosystem impacts. Previous studies have already shown that there can be considerable spatial overlap between foraging areas and fishing hotspots for a number of krill predators (11, 13–17). Point observations have further demonstrated that fishing vessels sometimes operate in close proximity to krill predators such as penguins and baleen whales (18), resulting in direct competition for krill and potential negative impacts to predators at local scales in krill poor years (12, 19).

The financial and logistical constraints of running fisheries and predator monitoring programmes in this vast and remote region currently limit the ability to detect ecosystem impacts, which is key to achieving the goals of ecosystem-based fisheries management. Therefore, cost-effective data collections with broad temporal and spatial coverage that can inform fisheries management are of high value.

## Study Concept.

In this study, we extracted encounters between krill fishing vessels and air-breathing krill predators from more than 30,000 h of acoustic data recorded with echosounders aboard three different krill fishing vessels (FV Antarctic Endurance, FV Antarctic Sea, and FV Saga Sea) from six fishing seasons (2016–2018 and 2021–2023) and in all currently relevant spatial fishery management units (CCAMLR Subareas 48.1 to 48.3). We interpreted these events as a low-level indicator of the fishery’s broader interaction with the Southern Ocean ecosystem, since they represent periods during which vessels and key krill predators compete directly for the same resource. The dataset enabled us to identify the times and locations at which predator encounter rates peaked, as well as assessing their dynamics in light of changing fleet behavior. Finally, we evaluated the effectiveness of management measures introduced to protect penguin colonies during their breeding season by comparing penguin encounter rates before and after the measures were introduced.

The use of acoustic data from fishing vessels has the unique advantages of being a low-cost by-product of routine fishing operations, providing temporal and spatial coverage that would be difficult to obtain using traditional sampling platforms, and providing an information-rich archive of encounters between fishing vessels, krill, and krill predators from a first-hand perspective that has not yet been used for this purpose.

**Fig. 1.**
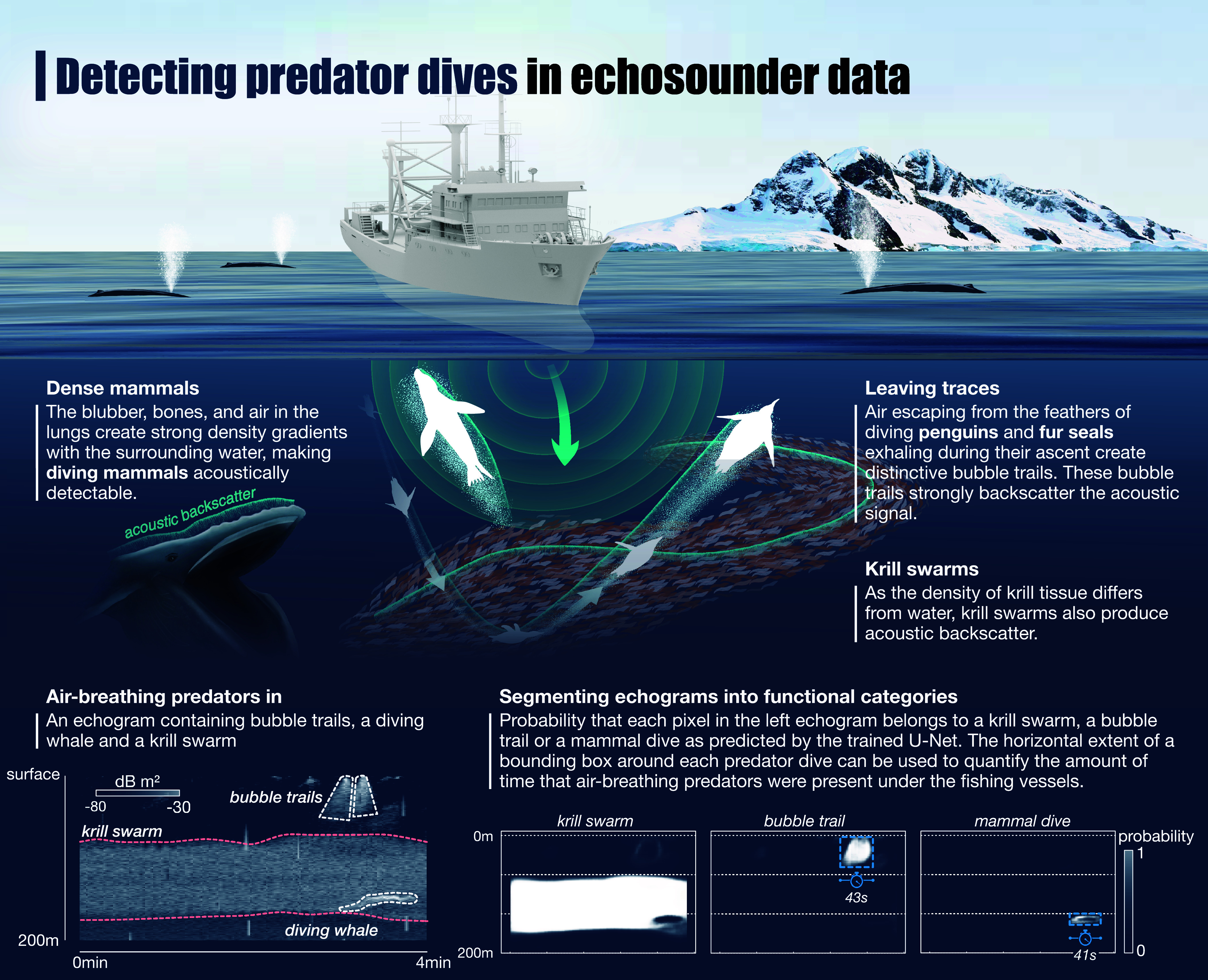
Conceptual basis for the detection of air-breathing predators in echosounder data. Movie S1 shows how bubble trails are produced in the acoustic data by diving penguins.

The fishery uses echosounders and sonars to detect krill swarms in the water column. The basic principle of echosounders is that matter of different density to the surrounding water cause emitted acoustic waves to be backscattered, which is in turn recorded by a detector mounted on the hull of the vessel. Therefore, not only krill swarms but also krill predators that dive below the fishing vessels produce backscatter that can be characterized as 1) comet-like streaks caused by bubble trails left by diving Antarctic fur seals during their ascent (20, 21) as well as penguins and; 2) intense signals caused by the massive bodies and the air trapped in the lungs of diving mammals [Fig. 1, (22)].

We trained a convolutional neural network [U-Net (23)] to identify these distinct signals (krill swarms, bubble trails, and diving mammals), and applied it to the acoustic data. To limit our analysis to periods when the vessels were actively fishing, we only analyzed periods when the Global Fishing Watch vessel activity classification algorithm (24) classified the FV Saga Sea and the FV Antarctic Endurance as “fishing.” Global Fishing Watch classifies fishing vessel status with >90% accuracy based on vessel position data obtained from the automatic identification system (AIS) using a convolutional neural network (25). Incorrectly set AISs could potentially lead to inaccuracies in the classification of vessel status. In the krill fishery, CCAMLR Conservation Measure 10-04 requires the fleet to report its exact position in near real time, and to our knowledge there are no known violations of this measure. In addition, Global Fishing Watch provides position data for the Norwegian krill fishing fleet based on AIS and Vessel Monitoring Systems (VMS), which is the system used to report vessel positions to CCAMLR. Comparison of vessel tracks based on VMS and AIS shows good agreement between the two systems, and we do not expect misreported vessel positions to have a major impact on the classification of vessel status.

Vessel status classification was not available for the FV Antarctic Sea and we filtered the data from this vessel based on a vessel speed window of 0.3 to 5 kn. This window excluded periods when the vessel was stationary (in port or during offloading operations) or steaming. More information on data processing and model application is provided in *Materials and Methods*.

Based on the temporal extent of the extracted predator dives, we derived the minutes of predator presence under the vessels, which we then normalized by the hours of available observations in time or space (Fig. 1). Although the three vessels in our analysis belonged to the Norwegian fishing company Aker BioMarine, we assume that the results are representative for the entire fishing fleet, which consists of around 10 vessels. This is due to the highly synchronized movement of krill fishing vessels, with median distances between vessels of <25 km in all six fishing seasons analyzed (*SI Appendix*, Fig. S5).

## Results and Discussion

### Classifying Mammal Dives.

Baleen whales (mainly fin and humpback whales) and Antarctic fur seals are the most abundant mammalian krill predators in the areas where krill fishing takes place. In addition, both taxonomic groups are known to interact with krill fishing vessels (18, 20) and are most likely responsible for the predator dives detected in the acoustic data. As whales and Antarctic fur seals have different ecological roles and life history strategies, classifying the predator dives to a taxonomic group level greatly improves the interpretation of our data.

**Fig. 2.**
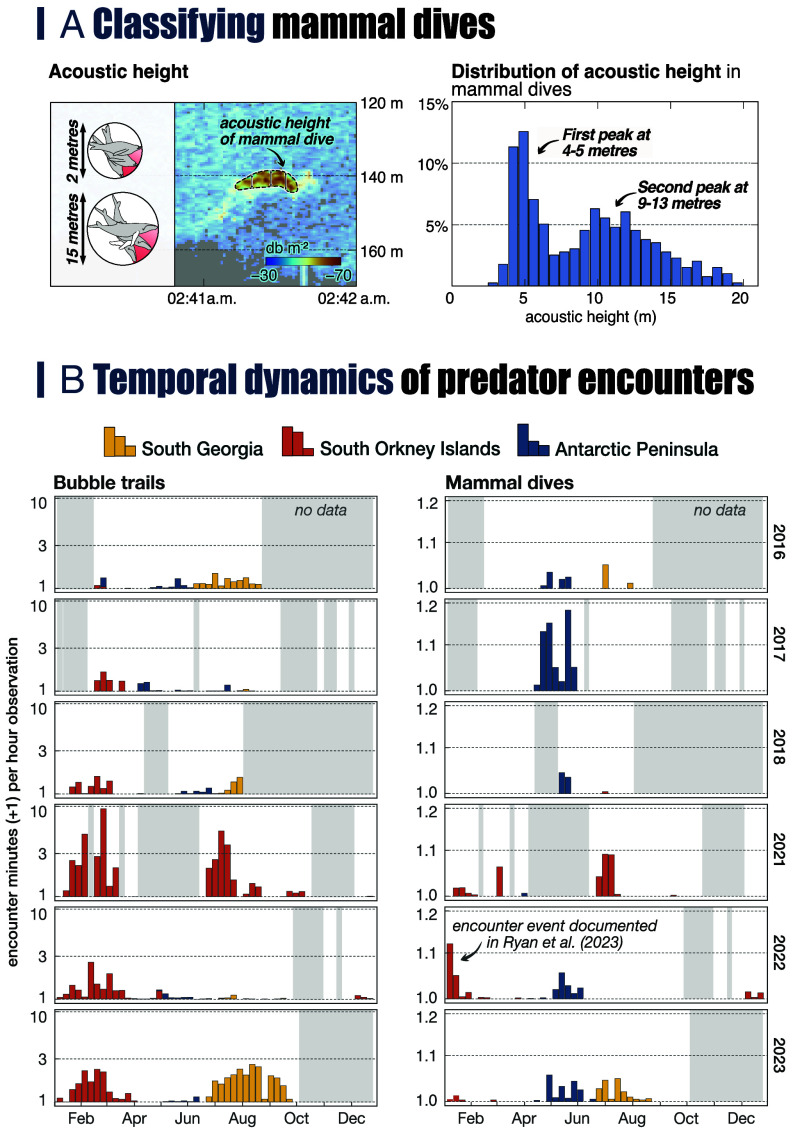
(*A*) Classifying mammal dives. *Left* panel: Acoustic height of an exemplary mammal dive. *Right* panel: Distribution of the acoustic height of detected mammal dives. (*B*) Temporal dynamics of encounters between krill fishing vessels and air-breathing predators from 2016 to 2018 and 2021 to 2023). Gray areas highlight periods where no observations were available. The y-scales are log10-scaled and 1 was added to the predator encounters to allow log-transformation when encounter rates were zero.

As the body dimensions of Antarctic fur seals and whales differ strongly, we expect the two predators to produce acoustic signals that reflect these differences, even after accounting for the angle of the predator’s body axis relative to the acoustic beam (Fig. 2*A*). Antarctic fur seals have a maximum body length of around 2 m, which means that the height of a seal signal should be a maximum of 2 m, but often less as it is unlikely that seals always dive perpendicular to the transducer. In contrast, baleen whales (humpback/fin whales) have a body diameter of 3 to 6 m (depending on the rotation along the longitudinal body axis) and average lengths of 15 to 25 m. The height of diving whale signals should therefore range between 3 m and more than 20 m.

In our data, the acoustic heights of extracted mammal dives peak at 4 to 5 m and 9 to 13 m (Fig. 2*A*), indicating that they predominantly show baleen whales. The lack of signals corresponding to the height of Antarctic fur seals is probably due to the vertical data resolution of 0.5 m, which means that the signal of a swimming seal is only a few pixels, making it difficult to detect robustly.

### Classifying Bubble Trails.

Classification of bubble trails to taxonomic group level (Antarctic fur seals, penguins) is more challenging without additional field observations. Both predator groups have been observed near and under krill fishing vessels [Movie S1, (20)] and have even been subject to net entanglements (26, 27).

However, in terms of the predominance of the two predator groups in the bubble trails detected, we expect clear regional differences. At the Antarctic Peninsula and South Orkney Islands, krill-dependent penguins (Chinstrap, Adélie and Gentoo penguins) vastly outnumber resident Antarctic fur seals [more than 2.8 million penguins (28) vs. less than 10,000 Antarctic fur seals (29) in Subarea 48.1, and almost 900,000 penguins (28) vs. around 20,000 Antarctic fur seals in Subarea 48.2, which includes subadult and male migrant seals from South Georgia that stay throughout late summer and early autumn (30, 31)]. In addition, the main fishing grounds at the South Orkney Islands and at the Antarctic Peninsula strongly overlap with the summer foraging grounds of several large penguin colonies (14, 32, 33). We therefore expect bubble trails at the South Orkney Islands and at the Antarctic Peninsula to be primarily caused by diving penguins, which is in line with our own observations (e.g. see Movie S1 which was filmed at the South Orkney Islands).

At South Georgia (Subarea 48.3), the situation is different with an Antarctic fur seal population of around 3.5 million (34) and approximately 1 to 2 million krill-dependent penguins [Gentoo, Chinstrap, and Macaroni penguins; (35)]. In addition, the main fishing grounds along the northwestern and northeastern shelf break (*SI Appendix*, Fig. S3) overlap with the foraging grounds of both, female Antarctic fur seals (36) and penguins (15) during winter. We therefore expect that both groups of predators contribute significantly to the bubble trails detected at South Georgia (20), and discuss our results accordingly.

### Predator-Vessel Encounters in Space and Time.

**Fig. 3.**
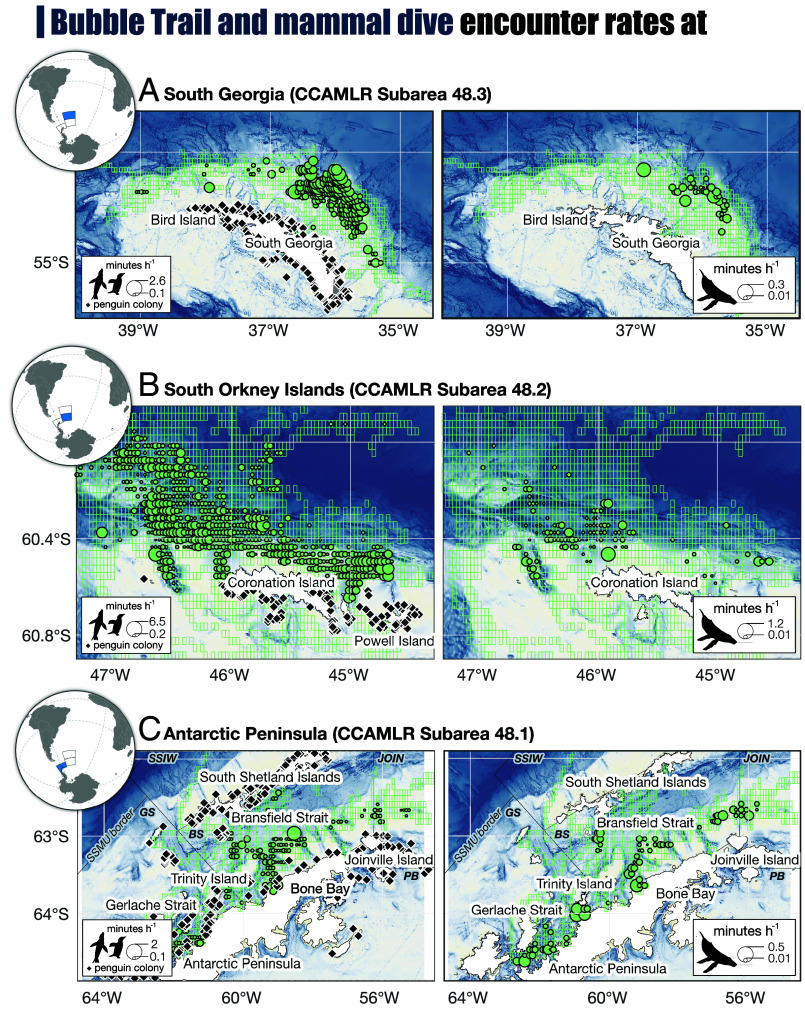
Mapping the spatial distribution of predator-fishery encounters at (*A*) South Georgia (grid resolution 0.04 × 0.04^°^), (*B*) South Orkney Islands (grid resolution 0.03 × 0.03^°^), and (*C*) the Antarctic Peninsula (grid resolution 0.075 × 0.075^°^) including the borders of proposed small scale management units (SSMU). Penguin colony locations from South Georgia GIS and MAPPPD (28). The green rectangles highlight all grid cells for which observations were available. Note that the point sizes scale differently across regions.

Mapping the predator encounters in Subareas 48.1 to 48.3 in space and time showed that a) predator encounters occurred year-round, b) there were predator-specific seasonal and regional encounter patterns (Fig. 2*B*), and c) the spatial distribution of predator encounters was often associated with topographic features such as shelf slopes or underwater canyons (Fig. 3).

In recent years, the fishery has typically started its fishing season in austral summer at the South Orkney Islands. Bubble trail encounter rates were high during this time, indicating that fishing vessels frequently encountered penguins at this time of year (Fig. 2*B*). Whale encounters were generally not frequent at this time, although one event, when the three fishing vessels from our data were observed fishing close to a feeding aggregation of fin and humpback whales in January 2022 (18), was associated with a sharp increase in whale encounters in our data at the same time (Fig. 2*B*).

Mapping the spatial distribution of penguin encounters showed that they were widespread across the South Orkney Islands shelf (Fig. 3*B*). A large underwater canyon north and northwest of Coronation Island, and a smaller canyon on-shelf west of Coronation Island could be identified as areas with high encounter rates for bubble trails and whales. The large canyon is known for its high density of krill during the summer (37, 38) and is an important summer foraging area for chinstrap penguins (14). The same canyon was also the site of a massive fin whale aggregation observed in 2022 (18). Some areas associated with high encounter rates north of Powell Island and west of Coronation Island have further been designated as Important Bird and Biodiversity Areas by BirdLife International based on a compilation of penguin tracking data (39); others, particularly those on the shelf north of Coronation Island, have not. In 2021, fishing vessels fished in the South Orkney Islands in winter, with predator encounter rates similar to or higher than those in summer (Fig. 2*B*). This highlights the importance of this region as a year-round foraging ground.

In late autumn, the fishing vessels usually moved to Subarea 48.1 at the Antarctic Peninsula, where fishing took place close to the coast and in Bransfield Strait. Bubble trail encounters in this region were rare whereas whale encounters were frequent compared to other regions. The rapid growth of regional humpback whale populations (40, 41) was not reflected in the whale encounter rates, which were relatively consistent across years (Fig. 2*B*). The lack of penguin encounters was surprising given their high regional abundance. One possible reason may be that many penguins disperse far from their colonies during autumn and winter, and those that remain in the wider area have often been reported along the shelf break west of the South Shetland Islands, as have the resident Antarctic fur seals (42, 43).

Areas of high whale encounter rates were generally associated with underwater canyons and coastal bays in the Gerlache Strait, south of Trinity Island, in Bone Bay, at the mouths of underwater canyons in the Bransfield Strait, and north of Joinville Island (Fig. 3*C*). These areas aligned well with important foraging areas of humpback whales in autumn (11, 16). However, the area north of Joinville Island has, to our knowledge, not been reported as an area of high fishery-predator overlap before. The general pattern of high whale but low penguin/seal encounter rates at the Antarctic Peninsula persisted into the winter before the vessels moved to either the South Orkney Islands or South Georgia.

The winter fishery at South Georgia was generally associated with high encounter rates of bubble trails, but interannual variability could be strong, e.g. almost no encounters in 2022 vs. many in 2023 (Fig. 2*B*). Temporospatial encounter patterns with whales matched those of penguins and/or Antarctic fur seals, suggesting that when encounter events occurred, a range of air-breathing krill predators were present. Almost all encounters at South Georgia were located in an area along the northeastern shelf break (Fig. 3*A*). This area has been previously identified as sensitive to spatial overlap between fishing vessels, gentoo penguins, and Antarctic fur seals during winter, especially in poor krill years (15, 36, 44), and it generally appears to be a persistent area of high krill predator presence (44, 45). Interestingly, other Antarctic fur seal winter foraging areas on the northwestern shelf break (36) were not associated with increased bubble trail encounter rates in our data, which may indicate interannual variability in the importance of this foraging area.

### Changes in Fleet Behavior and Implications for Predator Encounter Rates.

**Fig. 4.**
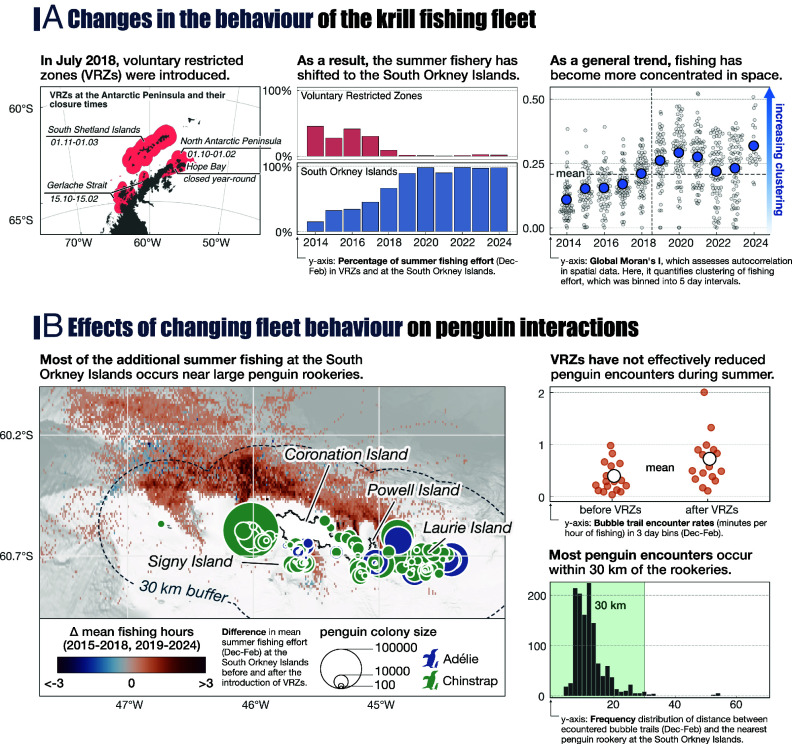
Changes in the behavior of fishing vessels and their impact on encounter rates with penguins in close proximity to key colonies. (*A*) Changes in the behavior of the krill fishing fleet since 2014. Movement data for the krill fishing vessels were obtained from Global Fishing Watch (24). (*B*) Increasing encounter rates with penguin colonies at the South Orkney Islands during summer. Summer fishing effort data were obtained from ref. 24, penguin colony data from ref. 28. The analyses comparing bubble trail encounter rates before and after the introduction of VRZs, as well as those examining the distance to the nearest colony, are based on observations from FV Saga Sea, since data from before 2018 were only available for this vessel. The green area in the nearest colony analysis shows the 30 km distance threshold.

Over the past decade, the behavior of the krill fishing fleet has shown two main trends: 1) fishing effort has become increasingly concentrated in space [Fig. 4*A*, (8)]; 2) since 2018, the fishery has largely stopped fishing at the Antarctic Peninsula in summer and instead shifted to the northern shelf areas of the South Orkney Islands (Fig. 4*A*). Although the regional shift is partly due to logistical and economic advantages [fishing in the South Orkney Islands reduces transit times for supply vessels, and fishing at the Antarctic Peninsula in autumn allows companies to harvest regional krill at its peak market value due to its high lipid content (46)], it is unintentionally reinforced by the establishment of several management measures: Fishing at South Georgia is closed during summer (47); the establishment of the South Orkney Islands Southern Shelf Marine Protected Area prohibits fishing in southern shelf regions of the South Orkney Islands (48), and in July 2018, the Association of Responsible Krill harvesting companies introduced voluntary restricted zones (VRZs), which close large parts of the North Antarctic Peninsula and the South Shetland Islands to fishing during the summer to protect penguin colonies during their breeding season (Fig. 4*A*). The VRZs were designed as buffer zones of 30 km around land, the approximate maximum foraging range of penguins during the breeding season.

Analyzing the fleet behavior at the South Orkney Islands during summer showed that: 1) much of the additional fishing effort has occurred within 30 km of large chinstrap penguin colonies to the west of Coronation Island; 2) most penguin encounters occurred within 15 km of the rookeries; and 3) penguin encounter rates have not decreased since the introduction of VRZs (Fig. 4*B*). This suggests that the fishing fleet, with its current behavior, has the potential to interact strongly with breeding penguins in this biologically important region, which is home to more than 1 million penguins (28) and other marine wildlife and krill-dependent predators (7, 13, 14). Since 2017, krill catches at the South Orkney Islands have increased by 100,000 tonnes, equivalent to 1 to 7% of the regional summer krill biomass (38) or the summer krill consumption of the entire breeding penguin population (assuming a demand of 100 kg krill per penguin as in ref. 32). As (voluntary) management measures applied independently at a regional level, VRZs and a summer closure at South Georgia have created a spatial management framework that relocates, but does not reduce, fishery-penguin co-occurrences.

### Informing Krill Fisheries Management.

With the presented analysis, we have been able to identify predator-specific periods and areas of increased fishery-predator overlap, including some not previously reported. Such findings not only improve our understanding of the distribution and persistence of different krill predator foraging grounds but can also inform future tagging and survey campaigns to obtain a more accurate picture of the temporospatial overlap of krill predators and the fishery.

Central place foragers such as Antarctic fur seals and penguins are often confined to colony-specific foraging grounds during their breeding season (45). The frequency with which individuals from specific predator colonies encounter fishing vessels is a metric that could help to identify the most impacted colonies and be included in survey data analyses. Remarkably, the CCAMLR Ecosystem Monitoring Programme (CEMP), which informs krill fisheries management, does not include and monitor the large chinstrap penguin colonies on Coronation Island, which are closest to the areas of greatest penguin-vessel encounters and the greatest increases in summer fishing effort (Fig. 4*B*).

In addition, our method allows for a rapid low-level assessment of the co-occurrence of predators and fishing vessels over large scales, which is important given the sometimes unpredictable nature of CCAMLR’s decision-making and fleet behavior. Surprisingly, the fishery did not start its 2025 season at the South Orkney Islands due to the presence of sea ice, but instead fished in the Gerlache Strait on the Antarctic Peninsula throughout December 2024 and early January 2025, violating the VRZs. With our method, we can quantitatively assess how such unexpected changes in fleet behavior affect fishery-predator encounters within days after each fishing season. This is particularly valuable in the current volatile period of CCAMLR, with unanimous decisions to introduce more sophisticated approaches to krill fisheries management in 2019, followed by an effective deregulation of the fishery in October 2024, and industry considerations to expand their operations into the Indian sector of the Southern Ocean, as described in Aker BioMarine’s Annual Report 2023, where predator monitoring programmes are not as well established. Finally, although baleen whales are the main predators of krill (32, 49) and are subject to fishery mortality (26), the impacts of the fishery on baleen whales is also not included in CEMP. With this in mind, data that can be systematically collected to improve our understanding of the competition and interactions between krill fishing vessels and baleen whales are important.

### Conclusions, Limitations, and Outlook.

Extracting predator dives from acoustic data from fishing vessels provided us with a comprehensive dataset for analyzing vessel-predator encounters in the krill fishery. We found that the South Orkney Islands, the new main fishing ground, stand out in terms of predator-fishery encounters. In addition, it is unlikely that independent regional conservation efforts reduced penguin-vessel encounters during the breeding season. Rather, these efforts created a framework that relocated penguin encounters to the South Orkney Islands. Our results emphasize that particular attention should be paid to investigating ecosystem dynamics at the South Orkney Islands if current fishing practices continue.

Despite the decisions made in October 2024, CCAMLR is currently revising its management approach for the krill fishery, and part of this revision includes the introduction of small-scale management units in Subarea 48.1, which would be used to allocate catches in space and time at small scales to minimize competition between krill-dependent predators and fishing vessels (50, 51). Small-scale management units have so far only been proposed for the CCAMLR Subarea 48.1 at the Antarctic Peninsula. Our results, and several other studies highlight the biological significance of the South Orkney Islands (14, 39, 42, 48), and it should be considered to introduce small-scale management units in all three CCAMLR Subareas, as has already been tested in several modeling studies (52–54).

In addition to these findings, our study demonstrates the high potential of using acoustic data from fishing vessels to inform fisheries management. In this study, we limited the data analysis to broad patterns of predator-vessel encounters, but other types of analysis focusing on smaller time scales, krill swarm behavior and distribution, or predator–prey interactions would be readily possible.

Although the acoustic data provide perspectives for assessing the ecosystem impacts of the krill fishery, there are some limitations to their interpretation. The actual impact of predator-vessel encounters depends on life stage and taxonomic group. For example, breeding penguins, which have limited flexibility in their foraging range, are disproportionately affected by local krill depletion and intensive fishing. In contrast, nonbreeding penguins and Antarctic fur seals, as well as baleen whales, would be able to roam freely, theoretically allowing them to change feeding grounds in the event of depletion, although this movement is associated with increased energetic costs of swimming and searching for prey.

Different species and populations of air-breathing krill predators, have different life history strategies and population states that need to be taken into account when interpreting the data. Ground-truthing and systematically comparing the extracted predator dives with predator tracking data (55), at-sea predator observations and predator distribution models will therefore be crucial to improve taxa classification and data interpretation. Our approach also only allows us to record predator encounters that occur directly below the fishing vessels, and predators that avoid these vessels but are in close proximity will be missed. For example, personal observations by the authors aboard commercial krill fishing vessels suggest that humpback whales were often found much closer to the vessels, sometimes even showing playful behavior, than fin whales, which typically kept their distance. Nevertheless, independently observed encounter events between krill predators and fishing vessels (18) were well reproduced by our data.

Finally, extracting predator dives from vessel-based acoustic data does not allow direct conclusions to be drawn on whether fishing is negatively affecting krill predator populations, highlighting the critical importance of predator monitoring programmes. However, it provides important quantitative information on predator-vessel encounters on an annual and regional scale. This information could be used to explain observed trends in predator populations and identify those populations and regions affected strongly by the fishery, with relatively little effort. Therefore, and despite their limitations, these data provide a valuable complementary perspective to traditional monitoring data.

## Materials and Methods

### Acoustic Data Access and Processing.

The acoustic data were recorded using ES60- and EK80 echosounders (Kongsberg Maritime AS), equipped with single-beam transducers operating at 38 kHz and one of 120 kHz or 200 kHz. Access to the raw acoustic data was provided by HUB Ocean via the Ocean Data Platform. To improve data handling, we first converted the available raw files into netcdf files using python and the krillscan (56) and echopype (57) packages. Old raw files that were recorded with ES60 echosounders were converted to netcdf files using the Large Scale Survey System (LSSS) (58). During file conversion, the acoustic data were regridded to a uniform temporal resolution of 1 s and a depth resolution of 0.5 m. Volume backscatter values were linearly interpolated when the ping frequency was greater than 1 s. To achieve the best model performance, we standardized the temporal and vertical extent of the acoustic data to 10 min and 500 m prior to image segmentation. In many cases, the raw acoustic files contained varying periods of observation, ranging from a few minutes to several hours. If the raw files contained less than 10 min of data or had a depth range of less than 500 m, the remaining time and depth values were filled with NA values to keep the uniform time and depth range. If a raw file contained more than 10 min of data, the file was split into multiple 10 min snippets. This procedure resulted in more than 200,000 individual 10-min datasets, and we then converted the volume backscatter values from the 120 kHz or 200 kHz channel into grayscale values in a fixed range from −90 to −20 dB to give krill swarms and predator dives more prominence in the acoustic signal. The resulting echograms (600 × 1,000 pixels) were then used as input files for the U-net, which was trained and applied using the tensorflow (59) package in *python*.

All subsequent analysis was done in R (60) with help of the terra (61), tidyterra (62), tidyverse (63), and scico (64) packages.

### Quantifying Predator Encounter Rates.

We trained the U-Net (23) based on 1,174 manually annotated images. This set was randomly split into 821 training images and 353 validation images. The annotated images were composed of equal proportions from each of the three vessels to account for intervessel variability. Images were randomly selected from the available fishing seasons to minimize temporal bias. To strike a balance between having a representative subset of the data that the U-net was later confronted with, and having enough examples of each object class to achieve robust predictive performance, we added 105 echograms containing predator and whale dives to the training dataset as they would otherwise have been underrepresented.

To improve model robustness the training images were augmented using the python package albumentations by applying random crops, flips, rotations, and elastic transformations. This resulted in a training set of 5,244 images. After testing multiple architecture, the best performing U-Net was chosen: a MultiRes U-Net with 4 encoder–decoder levels, a 521 neuron bridge and 7,244,736 trainable parameters, using a kernel size of 256 × 256 and five classes. The U-Net was trained on a set of high-performance GPUs by minimizing the categorical cross entropy using an Adam optimizer over 100 epochs and batch size of 20 images, the best performing network was stored. The manually segmented echograms consisted of 87% background, 10% krill, 1% bubble trails, 0.002% whales, and 0.008% sea floor. To account for this class imbalance, whale segments were weighted 10:1 with respect to the other classes during training.

After training, the U-Net classified individual pixels of the echograms into one of five classes: background, seafloor, krill swarm, whales, and bubble trails. The U-net achieved an accuracy of 83% for bubble trails and 62% for whales (F1 scores of 0.79 and 0.61, respectively). Echogram background was segmented with an F1 score of 0.96, krill swarms 0.91, and sea floor 0.86, although those were not considered in our analysis. For precision–recall curves, see *SI Appendix*, Fig. S1. Therefore, our results tend to underestimate true encounter rates, particularly for whales, but still provide a robust picture of the broad underlying patterns. When we applied the U-net to the acoustic data, we assigned each pixel to one of the five classes based on the optimal threshold probabilities shown in *SI Appendix*, Fig. S1. When the echograms contained considerable noise or when the seafloor had complex structure, the U-net could produce relatively high rates of false positives for the two predator classes. Therefore, a control plot was created for each predator classification, which allowed us to manually remove false positives after segmentation.

To quantify the encounter rates between fishing vessels and air-breathing predators, we converted the cleaned U-net predictions into polygons outlining the individual bubble trails or mammal dives. By adding the horizontal extents of each predator polygon (unit in pixels) and converting them to units of time (1 pixel = 1 s), we obtained the number of minutes that predators spent under the fishing vessels in an echogram. To obtain predator encounter rates, we added these periods of predator presence over time or in spatial grid cells, and divided them by the number of hours of observations available.

### Characteristics of the Available Data.

The available data covered 6 fishing seasons from 2016 to 2018 and 2021 to 2023. The amount of observations varied between years and months and most observations were available for the period from 2021 to 2023 (*SI Appendix*, Fig. S2). The contribution of each vessel to the available data varied between months and years, and data from 2016 to 2018 were not available from the FV Antarctic Endurance, which was built in 2018. Despite these interannual variations, our analyses showed that the temporal dynamics of predator encounters were relatively robust between seasons and regions.

Most observations (13,883 h) were available for the South Orkney Islands, which is consistent with this region being the main fishing ground (*SI Appendix*, Fig. S3). South Georgia and the Antarctic Peninsula were represented with 8,256 and 8,617 h of data, respectively, and the local spatial distribution of the data reflected existing fishing hotspots (*SI Appendix*, Fig. S3).

With the exception of autumn 2016, the diel distribution of observations across seasons was relatively even in each year (*SI Appendix*, Fig. S4), minimizing the risk of sampling bias as the activity profiles of many krill predators show strong diel cycles (65–68).

## Supplementary Material

Appendix 01 (PDF)

Movie S1. Chinstrap penguins diving in front of *FV Antarctic Endurance*, producing bubble trails in the acoustic recordings. ©Sebastian Menze.

## Data Availability

Raw acoustic data; the trained U-Net and a documentation of the full data processing and analysis workflow, as well as the associated R and Python code data have been deposited in [HUBOcean; Zenodo] (https://app.hubocean.earth/catalog/collection/1e3401d4-9630-40cd-a9cf-d875cb310449-akbm-collection (69); DOI: 10.5281/zenodo.15032844) (70). All other data are included in the manuscript and/or supporting information.

## References

[1] FAO, Global capture production. FishStat. https://www.fao.org/fishery/en/collection/capture?lang=en. Accessed 26 February 2025.

[2] CCAMLR Secretariat, “Fishery Summary 2023: *Euphausia superba* in Area 48” (Tech. Rep., 2024).

[3] E. Murphy *et al*., Spatial and temporal operation of the Scotia Sea ecosystem: A review of large-scale links in a krill centred food web. *Philos. Trans. R. Soc. B Biol. Sci.* **362**, 113–148 (2007). 10.1098/rstb.2006.1957 PMC1764830 17405210

[4] T. Ballerini , Productivity and linkages of the food web of the southern region of the western Antarctic Peninsula continental shelf. Prog. Oceanogr. **122**, 10–29 (2014).

[5] R. Perissinotto, E. A. Pakhomov, C. D. McQuaid, P. W. Froneman, In situ grazing rates and daily ration of Antarctic krill *Euphausia superba* feeding on phytoplankton at the Antarctic Polar Front and the Marginal Ice Zone. Mar. Ecol. Prog. Ser. **160**, 77–91 (1997).

[6] K. L. Haberman, R. M. Ross, L. B. Quetin, Diet of the Antarctic krill (*Euphausia superba* Dana): II. Selective grazing in mixed phytoplankton assemblages. J. Exp. Mar. Biol. Ecol. **283**, 97–113 (2003).

[7] H. Herr *et al*., Return of large fin whale feeding aggregations to historical whaling grounds in the Southern Ocean. *Sci. Rep.* **12**, 9458 (2022). 10.1038/s41598-022-13798-7 PMC9262878 35798799

[8] F. Santa Cruz, B. Ernst, J. A. Arata, C. Parada, Spatial and temporal dynamics of the Antarctic krill fishery in fishing hotspots in the Bransfield Strait and South Shetland Islands. Fish. Res. **208**, 157–166 (2018).

[9] R. D. Cavanagh , Future risk for southern ocean ecosystem services under climate change. Front. Mar. Sci. **7**, 615214 (2021).

[10] S. L. Hill *et al*., Is current management of the Antarctic krill fishery in the Atlantic sector of the Southern Ocean precautionary? *CCAMLR Sci.* **23**, 31–51 (2016).

[11] R. R. Reisinger , Spatiotemporal overlap of baleen whales and krill fisheries in the Western Antarctic Peninsula region. Front. Mar. Sci. **9**, 914726 (2022).

[12] G. M. Watters, J. T. Hinke, C. S. Reiss, Long-term observations from Antarctica demonstrate that mismatched scales of fisheries management and predator-prey interaction lead to erroneous conclusions about precaution. *Sci. Rep.* **10**, 2314 (2020). 10.1038/s41598-020-59223-9 PMC7012885 32047241

[13] A. D. Lowther, I. Staniland, C. Lydersen, K. M. Kovacs, Male Antarctic fur seals: Neglected food competitors of bioindicator species in the context of an increasing Antarctic krill fishery. *Sci. Rep.* **10**, 18436 (2020). 10.1038/s41598-020-75148-9 PMC7595138 33116190

[14] V. Warwick-Evans *et al*., Using habitat models for chinstrap penguins *Pygoscelis antarctica* to advise krill fisheries management during the penguin breeding season. *Divers. Distrib.* **24**, 1756–1771 (2018), https://onlinelibrary.wiley.com/doi/pdf/10.1111/ddi.12817.

[15] N. Ratcliffe *et al*., Changes in prey fields increase the potential for spatial overlap between gentoo penguins and a krill fishery within a marine protected area. *Divers. Distrib.* **27**, 552–563 (2021), https://onlinelibrary.wiley.com/doi/pdf/10.1111/ddi.13216.

[16] B. G. Weinstein, M. Double, N. Gales, D. W. Johnston, A. S. Friedlaender, Identifying overlap between humpback whale foraging grounds and the Antarctic krill fishery. Biol. Conserv. **210**, 184–191 (2017).

[17] J. A. Santora, R. R. Veit, C. S. Reiss, I. D. Schroeder, M. Mangel, Ecosystem oceanography of seabird hotspots: Environmental determinants and relationship with Antarctic krill within an important fishing ground. Ecosystems **20**, 885–903 (2017).

[18] C. Ryan *et al*., Commercial krill fishing within a foraging supergroup of fin whales in the Southern Ocean. *Ecology* **104**, e4002 (2023), https://onlinelibrary.wiley.com/doi/pdf/10.1002/ecy.4002. 10.1002/ecy.4002 36807151

[19] L. Krüger, M. F. Huerta, F. Santa Cruz, C. A. Cárdenas, Antarctic krill fishery effects over penguin populations under adverse climate conditions: Implications for the management of fishing practices. Ambio **50**, 560–571 (2021). 32979187 10.1007/s13280-020-01386-w PMC7882667

[20] T. Ichii , Body length-dependent diel vertical migration of Antarctic krill in relation to food availability and predator avoidance in winter at South Georgia. Mar. Ecol. Prog. Ser. **654**, 53–63 (2020).

[21] S. K. Hooker *et al*., Fur seals do, but sea lions don’t - cross taxa insights into exhalation during ascent from dives. *Philos. Trans. R. Soc. B Biol. Sci.* **376**, 20200219 (2021). 10.1098/rstb.2020.0219 PMC8200655 34121462

[22] P. Annasawmy, J. K. Horne, C. S. Reiss, G. J. Macaulay, Characterizing Antarctic air-breathing predator dive patterns on a common prey base from stationary echosounders. Polar Sci. **39**, 100974 (2024).

[23] O. Ronneberger, P. Fischer, T. Brox, “U-Net: Convolutional networks for biomedical image segmentation” in *Medical Image Computing and Computer-Assisted Intervention - MICCAI 2015*, N. Navab, J. Hornegger, W. M. Wells, A. F. Frangi, Eds. (Springer International Publishing, Cham, 2015), pp. 234–241.

[24] G. F. Watch, *Transparency for a Sustainable Ocean* (Global Fishing Watch, 2024).

[25] D. A. Kroodsma *et al*., Tracking the global footprint of fisheries. *Science* **359**, 904–908 (2018). 10.1126/science.aao5646 29472481

[26] CCAMLR, Summary of incidental mortality associated with fishing activities collected in scientific observer and vessel data during the 2020 and 2021 seasons (2021). https://meetings.ccamlr.org/en/wg-fsa-2021/04-rev-1 (Accessed 6 February 2025).

[27] R. Crawford , Tangled and drowned: A global review of penguin bycatch in fisheries. Endanger. Species Res. **34**, 373–396 (2017).

[28] G. R. W. Humphries , Mapping Application for Penguin Populations and Projected Dynamics (MAPPPD): Data and tools for dynamic management and decision support. Polar Rec. **53**, 160–166 (2017).

[29] D. J. Krause *et al*., Evaluating threats to South Shetland Antarctic fur seals amidst population collapse. *Mammal Rev.* **54**, 30–46 (2024), https://onlinelibrary.wiley.com/doi/pdf/10.1111/mam.12327.

[30] C. M. Waluda, S. Gregory, M. J. Dunn, Long-term variability in the abundance of Antarctic fur seals *Arctocephalus gazella* at Signy Island, South Orkneys. Polar Biol. **33**, 305–312 (2010).

[31] A. R. Carlini, G. A. Daneri, R. Casaux, M. E. I. Márquez, Haul-out pattern of itinerant male Antarctic fur seals (*Arctocephalus gazella*) at Laurie Island, South Orkney Islands. *Polar Res.* **25**, 139–144 (2006).

[32] V. Warwick-Evans *et al*., Using seabird and whale distribution models to estimate spatial consumption of krill to inform fishery management. *Ecosphere* **13**, e4083 (2022), https://onlinelibrary.wiley.com/doi/pdf/10.1002/ecs2.4083.

[33] J. J. Freer *et al*., A new dynamic distribution model for Antarctic krill reveals interactions with their environment, predators, and the commercial fishery in the south Scotia Sea region. *Limnol. Oceanogr.* **70**, 833–849 (2025), https://onlinelibrary.wiley.com/doi/pdf/10.1002/lno.12809.

[34] J. Forcada *et al*., Ninety years of change, from commercial extinction to recovery, range expansion and decline for Antarctic fur seals at South Georgia. *Glob. Chang. Biol.* **29**, 6867–6887 (2023), https://onlinelibrary.wiley.com/doi/pdf/10.1111/gcb.16947. 10.1111/gcb.16947 37839801

[35] A. Clarke, J. P. Croxall, S. Poncet, A. R. Martin, R. Burton, Important bird areas: South Georgia. Br. Birds **105**, 118–144 (2012).

[36] C. C. G. Bamford, V. Warwick-Evans, I. J. Staniland, J. A. Jackson, P. N. Trathan, Wintertime overlaps between female Antarctic fur seals (*Arctocephalus gazella*) and the krill fishery at South Georgia, South Atlantic. *PLoS One* **16**, e0248071 (2021). 10.1371/journal.pone.0248071 PMC7932113 33662029

[37] B. A. Krafft , Summer distribution and demography of Antarctic krill *Euphausia superba* Dana, 1850 (Euphausiacea) at the South Orkney Islands, 2011–2015. J. Crustac. Biol. **38**, 682–688 (2018).

[38] G. Skaret , Distribution and biomass estimation of Antarctic krill (*Euphausia superba*) off the South Orkney Islands during 2011–2020. ICES J. Mar. Sci. **80**, 1472–1486 (2023).

[39] M. P. Dias *et al*., Identification of marine Important Bird and Biodiversity Areas for penguins around the South Shetland Islands and South Orkney Islands. *Ecol. Evol.* **8**, 10520–10529 (2018), https://onlinelibrary.wiley.com/doi/pdf/10.1002/ece3.4519. 10.1002/ece3.4519 PMC6238121 30464824

[40] L. J. Pallin , High pregnancy rates in humpback whales (*Megaptera novaeangliae*) around the Western Antarctic Peninsula, evidence of a rapidly growing population. R. Soc. Open Sci. **5**, 180017 (2018). 29892441 10.1098/rsos.180017 PMC5990787

[41] L. J. Pallin , A surplus no more? Variation in krill availability impacts reproductive rates of Antarctic baleen whales. Glob. Chang. Biol. **29**, 2108–2121 (2023). 36644792 10.1111/gcb.16559

[42] J. T. Hinke *et al*., Identifying Risk: Concurrent overlap of the Antarctic krill fishery with krill-dependent predators in the Scotia Sea. *PLoS One* **12**, e0170132 (2017). 10.1371/journal.pone.0170132 PMC5234819 28085943

[43] J. T. Hinke, M. M. Santos, M. Korczak-Abshire, G. Milinevsky, G. M. Watters, Individual variation in migratory movements of chinstrap penguins leads to widespread occupancy of ice-free winter habitats over the continental shelf and deep ocean basins of the Southern Ocean. *PLoS One* **14**, e0226207 (2019). 10.1371/journal.pone.0226207 PMC6903731 31821380

[44] K. A. Owen , At-sea distribution of marine predators around South Georgia during austral winter, with implications for fisheries management. Polar Biol. **47**, 663–679 (2024).

[45] I. Boyd, I. Staniland, A. Martin, Distribution of foraging by female Antarctic fur seals. Mar. Ecol. Prog. Ser. **242**, 285–294 (2002).

[46] N. Hellessey , Seasonal and interannual variation in the lipid content and composition of *Euphausia superba* Dana, 1850 (Euphausiacea) samples derived from the Scotia Sea fishery. J. Crustac. Biol. **38**, 673–681 (2018).

[47] P. N. Trathan *et al*., “Chapter two - the South Georgia and the South Sandwich Islands MPA: Protecting A biodiverse Oceanic Island chain situated in the flow of the Antarctic circumpolar current” in *Advances in Marine Biology, Marine Managed Areas and Fisheries*, M. L. Johnson, J. Sandell, Eds. (Academic Press, 2014), vol. 69, pp. 15–78. 10.1016/B978-0-12-800214-8.00002-5 25358297

[48] P. N. Trathan, S. M. Grant, “Chapter 4 - the South Orkney Islands Southern Shelf Marine protected area: Towards the establishment of marine spatial protection within international waters in the Southern Ocean” in *Marine Protected Areas*, J. Humphreys, R. W. E. Clark, Eds. (Elsevier), pp. 67–98 (2020).

[49] M. Biuw , Estimated summer abundance and krill consumption of fin whales throughout the Scotia Sea during the 2018/2019 summer season. Sci. Rep. **14**, 7493 (2024). 38553485 10.1038/s41598-024-57378-3 PMC10980806

[50] CCAMLR, Report of the Twenty-First Meeting of the Commission (CCAMLR-XXI) (2002).

[51] CCAMLR, Report of the Thirty-eighth Meeting of the Commission, CCCAMLR-38, Paragraphs 5.8 to 5.9 and Paragraphs 5.17 to 5.20 CCAMLR (2019).

[52] É. E. Plagányi, D. S. Butterworth, The Scotia Sea krill fishery and its possible impacts on dependent predators: Modeling localized depletion of prey. *Ecol. Appl.* **22**, 748–761 (2012), https://onlinelibrary.wiley.com/doi/pdf/10.1890/11-0441.1. 10.1890/11-0441.1 22645808

[53] G. M. Watters, S. L. Hill, J. T. Hinke, J. Matthews, K. Reid, Decision-making for ecosystem-based management: Evaluating options for a krill fishery with an ecosystem dynamics model. *Ecol. Appl.* **23**, 710–725 (2013), https://onlinelibrary.wiley.com/doi/pdf/10.1890/12-1371.1. 10.1890/12-1371.1 23865224

[54] E. S. Klein, G. M. Watters, Comparing feedback and spatial approaches to advance ecosystem-based fisheries management in a changing Antarctic. PLoS One **15**, e0231954 (2020). 32898163 10.1371/journal.pone.0231954 PMC7478840

[55] W. C. Oosthuizen *et al*., The foraging behavior of nonbreeding Adélie penguins in the western Antarctic Peninsula during the breeding season. *Ecosphere* **13**, e4090 (2022), https://onlinelibrary.wiley.com/doi/pdf/10.1002/ecs2.4090.

[56] S. Menze, G. J. Macaulay, G. Zhang, A. D. Lowther, B. A. Krafft, KRILLSCAN: An automated open-source software for processing and analysis of echosounder data from the Antarctic krill fishery. Fish. Manage. Ecol. **32**, e12739 (2025).

[57] W. J. Lee *et al*., A Python library for interoperable and scalable processing of water column sonar data for biological information. aRxiv [Preprint] (2021). http://arxiv.org/abs/2111.00187 (Accessed 15 January 2024).

[58] R. Korneliussen *et al*., “The large scale survey system - LSSS” in *Proceedings of the 29th Scandinavian Symposium on Physical Acoustics* (2006).

[59] M. Abadi *et al.*, “Tensorflow: Large-scale machine learning on heterogeneous distributed systems.” arXiv [Preprint] (2016). https://arxiv.org/abs/1603.04467 (Accessed 5 November 2023).

[60] R Core Team, R: A language and environment for statistical computing (R Version 4.3.0, R Foundation for Statistical Computing, Vienna, Austria, 2021).

[61] R. J. Hijmans, *terra*. R package version 1.7-46. https://github.com/rspatial/terra. Accessed 27 September 2022.

[62] D. Hernangómez, Using the tidyverse with terra objects: the tidyterra package. JOSS **8**, 5751 (2023).

[63] H. Wickham , Welcome to the Tidyverse. J. Open Source Softw. **4**, 1686 (2019).

[64] T. Pedersen, F. Crameri, scico: Colour Palettes Based on the Scientific Colour-Maps. R package version 1.5.0.9000. https://github.com/thomasp85/scico. Accessed 10 December 2023.

[65] A. S. Friedlaender, R. B. Tyson, A. K. Stimpert, A. J. Read, D. P. Nowacek, Extreme diel variation in the feeding behavior of humpback whales along the western Antarctic Peninsula during autumn. Mar. Ecol. Prog. Ser. **494**, 281–289 (2013).

[66] T. D. Williams, D. R. Briggs, J. P. Croxall, Y. Naito, A. Kato, Diving pattern and performance in relation to foraging ecology in the Gentoo penguin, *Pygoscelis papua*. *J. Zool.* **227**, 211–230 (1992), https://onlinelibrary.wiley.com/doi/pdf/10.1111/j.1469-7998.1992.tb04818.x.

[67] J. K. Jansen, P. L. Boveng, J. L. Bengtson, Foraging modes of chinstrap penguins: Contrasts between day and night. Mar. Ecol. Prog. Ser. **165**, 161–172 (1998).

[68] R. P. Wilson, B. Culik, N. R. Coria, D. Adelung, H. J. Spairani, Foraging rhythms in Adélie penguins (*Pygoscelis adeliae*) at hope bay, Antarctica; determination and control. Polar Biol. **10**, 161–165 (1989).

[69] HUB Ocean, Data from: “Aker BioMarine EK60, EK80 Echosounder data.” Ocean Data Platform, https://app.hubocean.earth/catalog/collection/1e3401d4-9630-40cd-a9cf-d875cb310449-akbm-collection. Deposited 30 October 2023.

[70] D. Bahlburg, S. Menze, Code and data for “Mapping encounters between Antarctic krill fishing vessels and air-breathing krill predators using acoustic data from the fishery”, Zenodo. https://zenodo.org/records/15032844. Deposited 9 May 2025. 10.1073/pnas.2417203122 PMC12207417 40523191

