# Supplementary material for: Mapping encounters between Antarctic krill fishing vessels and air-breathing krill predators using acoustic data from the fishery
Source: Proc Natl Acad Sci U S A. 2025 Jun 16;122(25):e2417203122. doi: 10.1073/pnas.2417203122 (PMC12207417; doi:10.1073/pnas.2417203122)
Supplement: Supplementary file 1 — Appendix 01 (PDF) [file pnas.2417203122.sapp.pdf]

Supporting Information for

Mapping interactions between Antarctic krill fishing vessels and air-breathing krill predators using acoustic data from the fishery

Dominik Bahlburg, Sebastian Menze, Bjørn A. Krafft, Andy D. Lowther, Bettina Meyer

Dominik Bahlburg.
E-mail: dominik.bahlburg1@awi.de

This PDF file includes:

Figs. S1 to S5
Legend for Movie S1

Other supporting materials for this manuscript include the following:

Movie S1

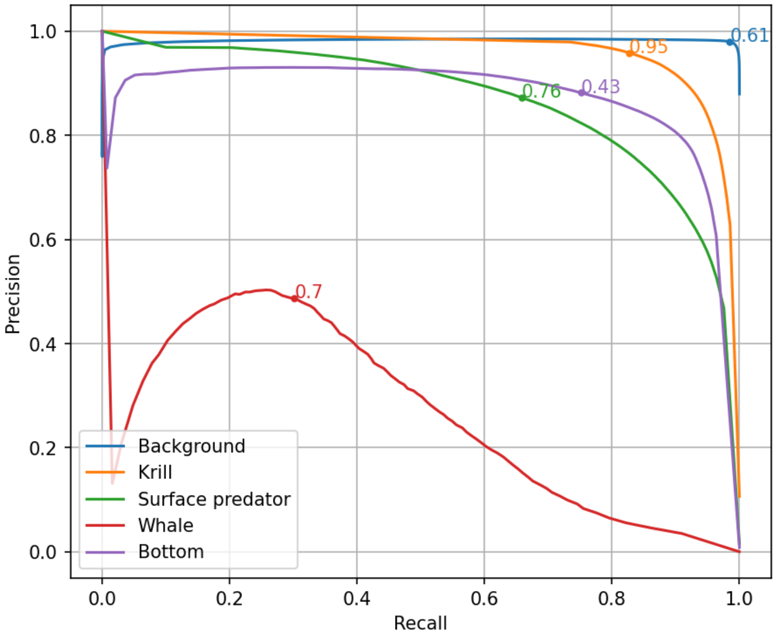

Fig. S1. Precision-recall curves for the trained U-net with threshold probabilities in coloured font used to extract both types of predators (surface predator = bubble trails, whale = whale/seal blob) from model predictions).

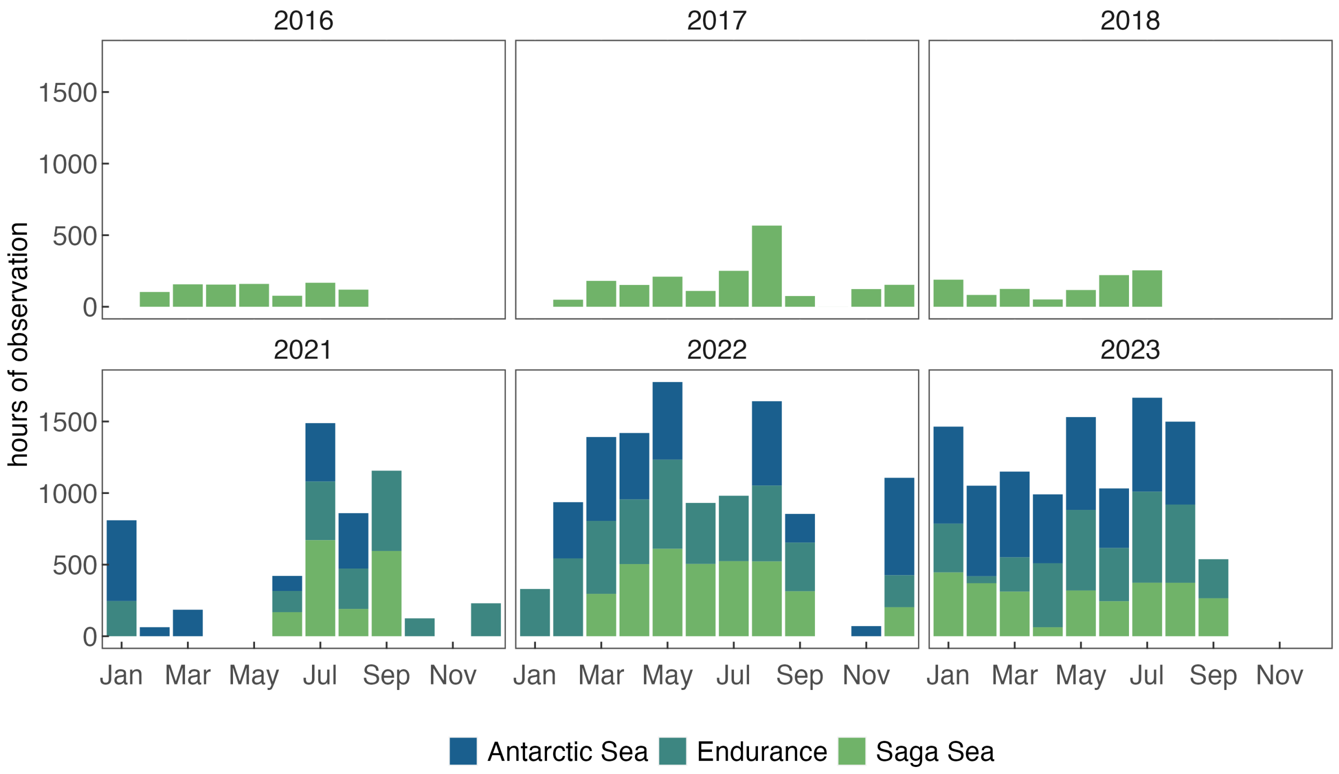

Fig. S2. Distribution of the available acoustic data over year, months and vessels.

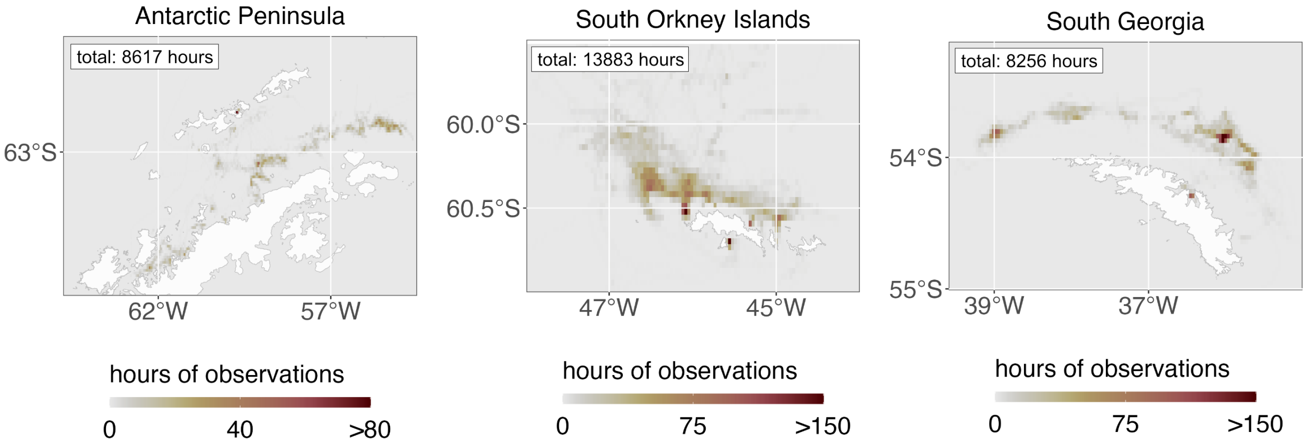

Fig. S3. Temporo-spatial distribution of the available observations.

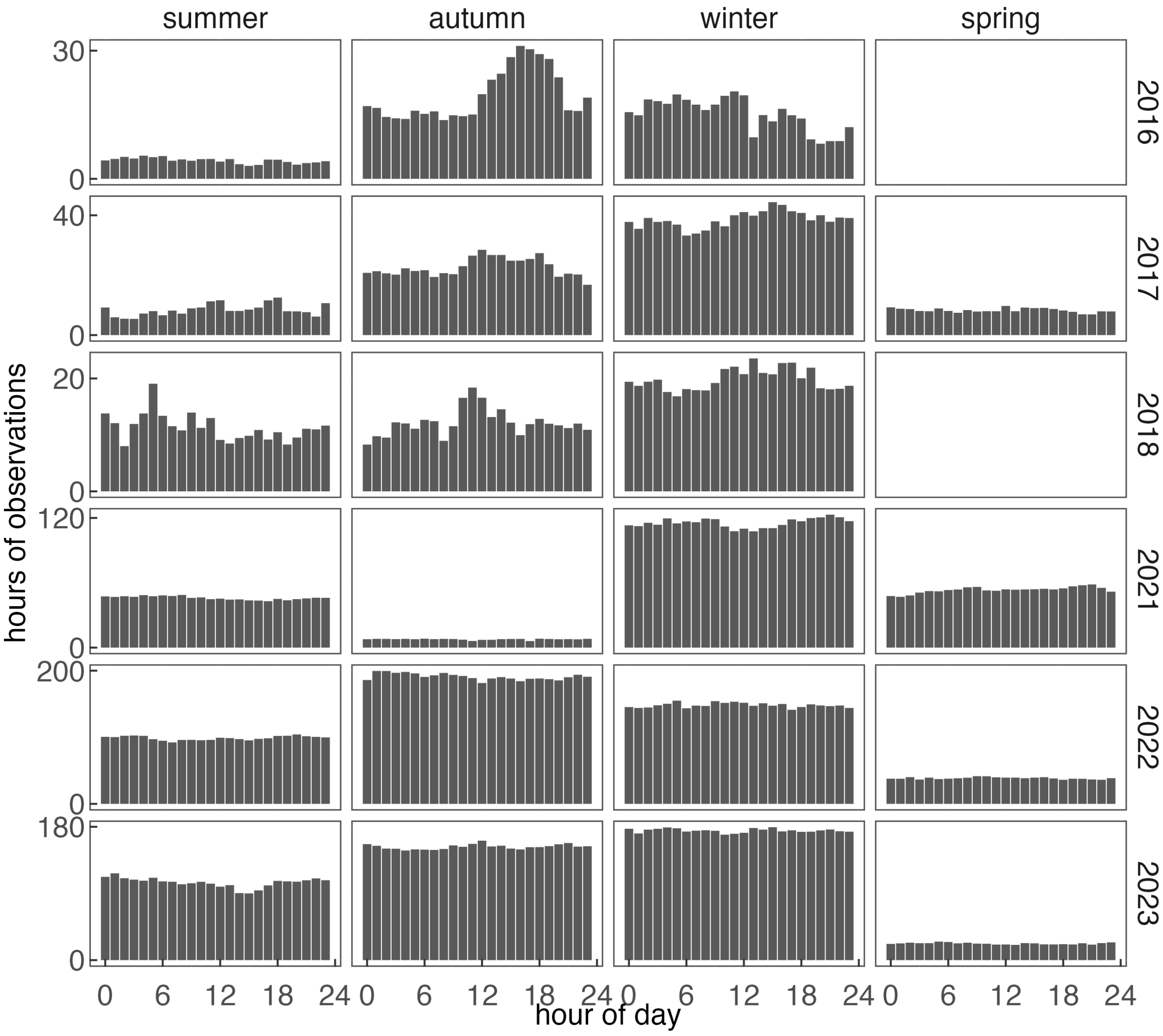

Fig. S4. Diel distribution of observations across seasons and years. Seasons were defined as follows: summer: November-January, autumn: February-April.

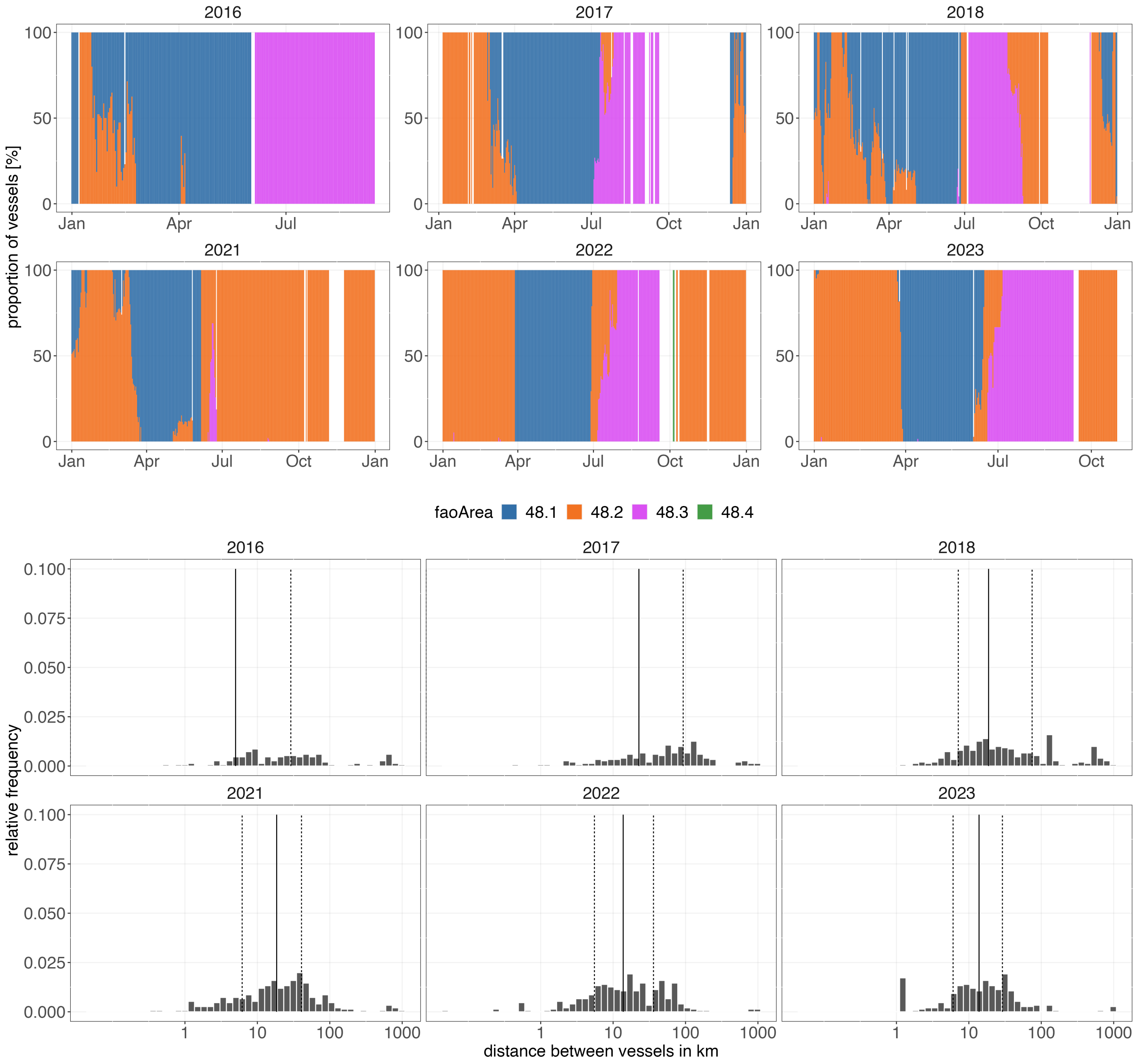

Fig. S5. a) Percentage of vessels in the total krill fishing fleet that fished in a given CCAMLR sub-area in 2021-2023. Data obtained via *globalfishingwatch*. b) Mean distance between vessels during 2021-2023. High values indicate that the fleet was spread over large areas. This typically occurs when individual vessels move to another fishing area to explore fishing conditions. Sometimes these vessels returned to their original location (e.g. in June 2021) and sometimes they were followed by the others (e.g. in July 2022).

Movie S1. Chinstrap penguins diving in front of *FV Antarctic Endurance*, producing bubble trails in the acoustic recordings. ©Sebastian Menze.
